# Atraumatic Spontaneous Transvaginal Small Bowel Evisceration: A Rare Surgical Emergency

**DOI:** 10.7759/cureus.56564

**Published:** 2024-03-20

**Authors:** Kevin J Fuentes, Sara Fernanda Arechavala Lopez, Irving Fuentes, Jorge Leal Hidalgo, Juan Jacobo Martínez Zarate

**Affiliations:** 1 General Surgery, Médica Sur, Mexico City, MEX; 2 Medicine, Universidad Autónoma Metropolitana, Mexico City, MEX; 3 Surgery, Médica Sur, Mexico City, MEX

**Keywords:** evisceration, transvaginal, ileum, postmenopausal women, hysterectomy

## Abstract

Abdominal pain ranks as the predominant cause for emergency department consultations. Although rare, transvaginal evisceration of the small intestine necessitates immediate surgical intervention due to its potential to induce intestinal ischemia and peritonitis. Key risk factors include postmenopausal status, a history of gynecologic surgery, and heightened abdominal pressure. Clinical presentation typically involves pain and protrusion of intestinal contents or even abdominal viscera. Diagnosis relies on thorough clinical assessment, and treatment strategies should be tailored to each patient. Here, we describe the case of a 65-year-old female patient with a non-traumatic evisceration of the ileum, who had undergone total abdominal hysterectomy following anterior colpocele a year ago, subsequently necessitating exploratory laparotomy and repair of the vaginal ampulla.

## Introduction

Abdominal pain constitutes the predominant reason for presentation in emergency departments across the United States, representing 6.8% of all visits [1]. Common etiologies encompass appendicitis, cholecystitis, ulcers, perforation, small intestine or colon obstruction, alongside other less prevalent causes. The transvaginal evisceration of the small intestine is a rare complication of gynecologic surgery, typically arising in conjunction with trauma or conditions heightening intra-abdominal pressure [2]. Its incidence varies between 0.032% and 0.28% [3], often accompanied by complications such as intestinal ischemia, ileus, and peritonitis [4], with a mortality rate of 5.6% [5]. Nevertheless, when intestinal strangulation through the vagina occurs, morbidity escalates significantly, necessitating emergent surgery due to its potential for morbidity and mortality. In this instance, we present the case of a 65-year-old female patient with atraumatic ileal evisceration, who had previously undergone total abdominal hysterectomy due to anterior colpocele.

## Case presentation

**Figure 1:**
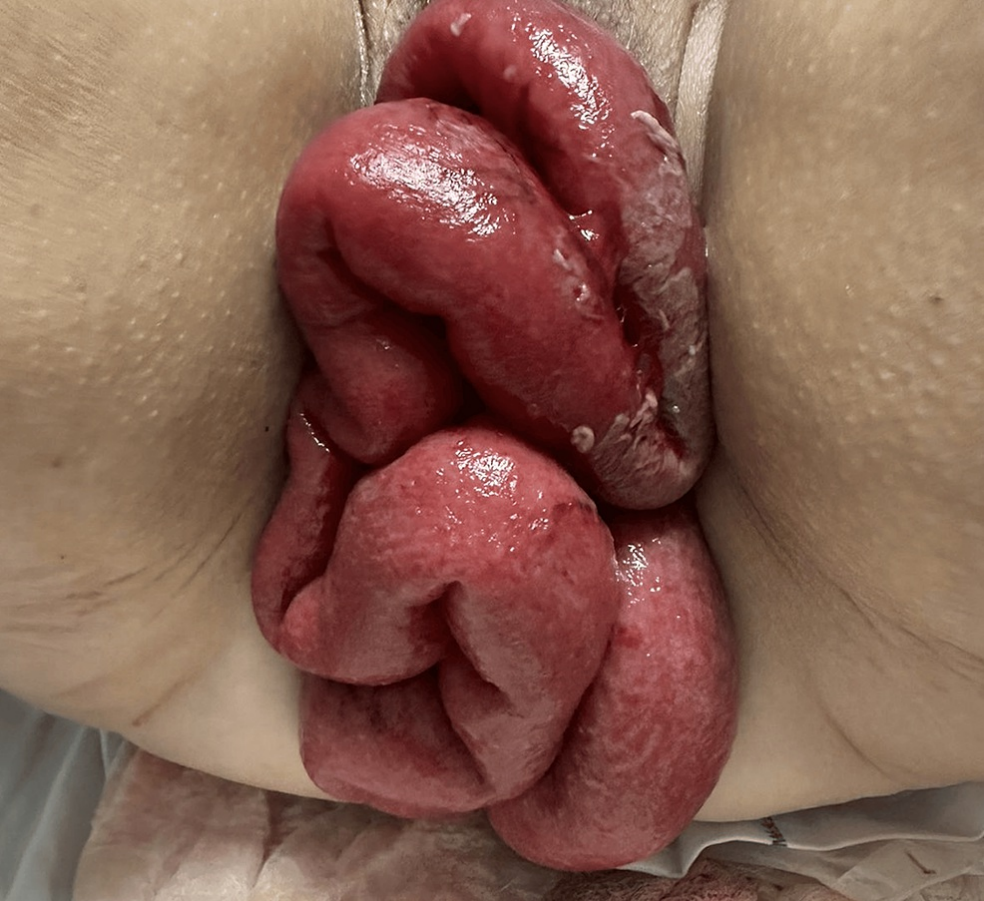
Protrusion of intestinal loops through the vaginal canal

A 65-year-old female, who had undergone a total abdominal hysterectomy secondary to anterior colpocele a year ago, presented to the emergency department. She reported experiencing abdominal pain after lifting a heavy weight (Valsalva maneuver), followed by the descent and externalization of intestinal loops through the vaginal canal. Upon physical examination, a protrusion of the small intestine through the vaginal canal with noticeable changes in coloration was observed (Figure 1).

**Figure 2:**
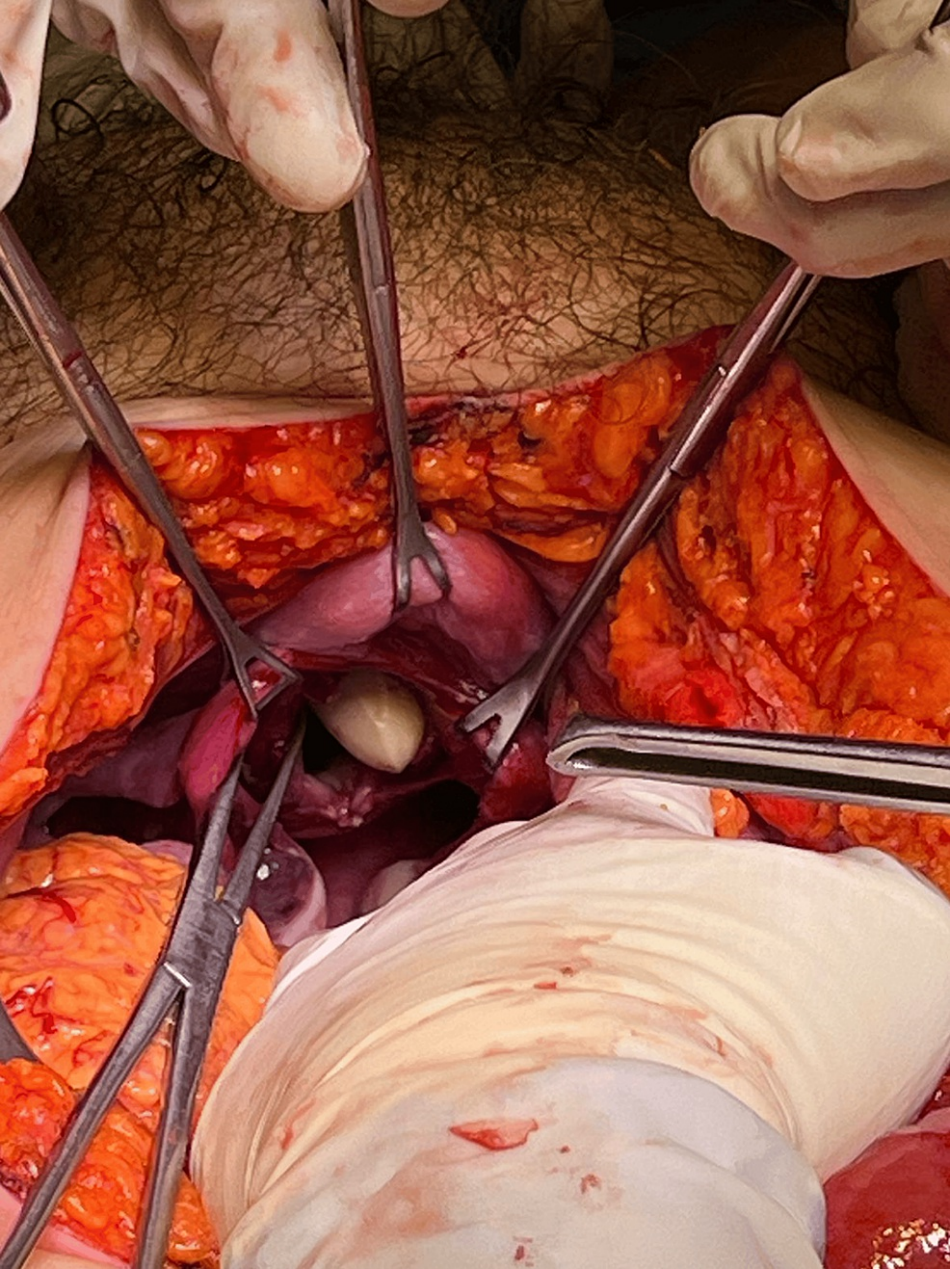
Vaginal examination showed a defect where the loops of the small intestine protruded

**Figure 3:**
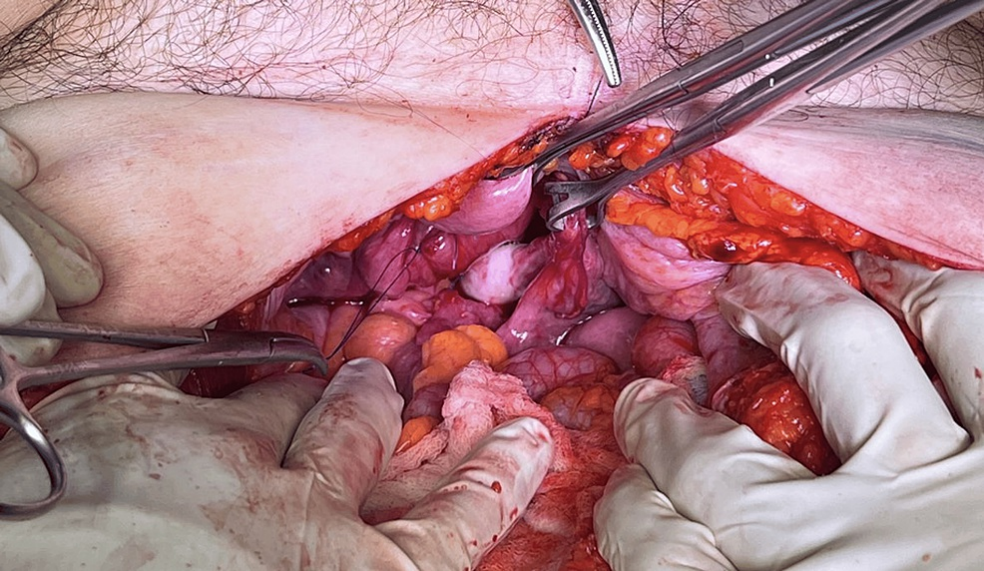
Reinforcement of the vaginal vault and closure of the peritoneum of the Douglas sac with non-absorbable suture

During her hospitalization, laboratory tests were conducted, yielding the following results: leukocytosis (10.29 x10^3/μL), increased CRP (97 mg/dL), and hyperlactatemia (2.2 mmol/L). The surgical team on call was consulted, who integrated the diagnosis of transvaginal evisceration, so, an exploratory laparotomy plus transvaginal hernia reduction and raffia of the vaginal ampule was performed (Figure 2). The reinforcement was carried out in two stages. Initially, the vaginal vault was strengthened using non-absorbable sutures followed by the closure of the peritoneum of the Douglas sac also with non-absorbable suture. Then, a vaginal examination was conducted to assess the closure of both planes (Figure 3).

The surgical findings revealed a 50 cm herniation of the terminal ileum with mesenteric torsion by default of 4x4 cm through the vaginal ampule. Subsequently, the closure of the planes was confirmed through a vaginal examination, and the surgical procedure concluded without any complications. Following the surgery, the patient experienced a satisfactory postoperative recovery. On the first day after the procedure, follow-up laboratory tests were conducted, revealing a decrease in leukocytes (10.08 x 10^3/μL), and lactate by 1.3 mmol/L, and although the CRP levels rose to 164 mg/dL, there was an improvement in symptoms, accompanied by satisfactory tolerance of the oral route. Furthermore, there was maintenance of adequate urinary volumes. Consequently, she was discharged without any complications. 

## Discussion

Hyernaux first described transvaginal evisceration in 1864 [6]. Around 75% of patients who present with it have a previous history of gynecologic surgery such as hysterectomy or vaginal prolapse repair [7]. The primary cause of transvaginal evisceration is typically attributed to the dehiscence of the vaginal vault, characterized by the rupture of the vaginal edges and the adjacent peritoneum [8], where evisceration may or may not occur. Transvaginal evisceration occurs more frequently in postmenopausal women, accounting for 70% of cases. This is attributed to the thinness, scarring, and reduced vascularity of the postmenopausal vagina, rendering it more susceptible to rupture. Notably, in postmenopausal women, vaginal ruptures typically manifest in the posterior fornix [9]. Some of the risk factors are postoperative infections, hematoma, smoking, prolonged use of steroids, diabetes mellitus, malnutrition, radiotherapy, chronic constipation, obesity, laparoscopic hysterectomy, and cervical cancer. Triggers include intercourse, straining during bowel movements, and increased intra-abdominal pressure during the Valsalva maneuver [10]. Croak et al. reported an incidence of vaginal evisceration of 0.032% following pelvic surgery, with an average occurrence at 27 months postoperatively [11]. In premenopausal women, risk factors for transvaginal evisceration include obstetric injury, vaginal infections, vaginal trauma, and unconventional sexual practices. Notably, engaging in sexual activities alone has been identified as a significant risk factor in this demographic [12].

The clinical presentation is sudden, characterized by abdominal or pelvic pain, accompanied by vaginal bleeding or not, and the protrusion of intestinal loops through the vagina. This occurrence is more frequently associated with the terminal ileum due to the length of the mesentery, its mobility, and its position. [13]. It may sometimes be accompanied by the omentum, fallopian tubes, cecal appendix, colon, and epiplastic appendages [14].

Diagnosis typically relies on clinical assessment, as direct visualization during physical examination confirms the condition, making imaging studies unnecessary. Treatment involves promptly administering intravenous antibiotics due to the extraperitoneal exposure of the intestine [15]. Hur et al. recommend attempting to reduce evisceration first, followed by inserting a bladder catheter to prevent urinary retention and packing the vagina [16]. Subsequently, manual reduction can be performed once intestinal viability is confirmed, and vaginal repair can be completed [17].

Currently, it is preferable to conduct a laparotomy, allowing the surgeon to perform an intra-abdominal inspection to confirm the integrity of the loops and assess for any compromise, along with ensuring adequate peritoneal lavage. Various approaches exist, including transvaginal, combined vaginal-abdominal, and laparoscopic-vaginal. The transvaginal approach is suitable for patients with an easily reducible, viable bowel and no signs of peritonitis. However, it should be avoided in cases of elevated vaginal defects or strangulated bowel. The combined vaginal-abdominal approach is recommended for patients with a strangulated yet viable bowel [18]. Regarding laparoscopic evisceration repair, the first documented case of laparoscopic repair for vaginal evisceration of the small intestine was published in 1996 [18]. The combined laparoscopic and transvaginal approach offers several advantages. Laparoscopy enables direct visualization and evaluation of the small intestine, facilitating the reduction of herniated intestinal contents. Simultaneously, the transvaginal approach ensures optimal closure of the vaginal stump.

One of the advantages of laparoscopy is its ability to circumvent the morbidity associated with laparotomy. Additionally, it entails shorter hospitalization times, facilitates gentle reduction procedures, and typically results in lower rates of postoperative pain. However, its main limitations include limited availability, higher costs, potential vascular compromise of the small intestine, and, above all, the requisite skill level of the surgeon. A direct visual and manual assessment of the defect and surrounding tissue is beneficial for determining the most suitable repair method. Sutures can be directly inserted through the vagina, which reduces surgical time and eliminates the need for costly laparoscopic suturing devices. Opting for open repair, with its inherent safety, can obviate the necessity for an omental patch and mitigate the potential risks associated with placing synthetic mesh. Concerning the closure of the defect at the vaginal vault level, in a series of cases, Matthews et al. suggested employing simple stitches using non-absorbable monofilament material [19]. This technique entails debriding the edges and integrating the vaginal mucosa with the pubocervical fascia and rectovaginal fascia, as described. We followed this approach in our patient, performing closure in two planes: initially reinforcing the vaginal vault with non-absorbable sutures and then closing the peritoneum of the Douglas pouch using non-absorbable sutures. Finally, a tactile assessment was conducted to ensure the closure of both planes.

Transvaginal evisceration may manifest from five days to 30 years post-gynecologic surgery. Recurrence rates, irrespective of the repair method, vary between 6% and 33% [19]. If left untreated, mortality rates can reach up to 10%, particularly in cases where there is evidence of intestinal compromise. Hence, prompt management is essential.

## Conclusions

Transvaginal evisceration is an exceptionally uncommon surgical emergency, with fewer than 100 reported cases documented worldwide. Key risk factors encompass postmenopausal status, a history of hysterectomy or gynecological surgery, and elevated intra-abdominal pressure. Clinically, patients usually present with pain and the protrusion of intestinal contents or viscera. Diagnosis primarily relies on clinical evaluation, and treatment strategies necessitate customization for each patient due to the wide range of therapeutic options available. However, despite weighing the risk-benefit ratio, laparotomy stands as the current preferred approach.
